# Supplementary material for: Identification and Analysis of WRKY Transcription Factors in Response to Cowpea Fusarium Wilt in Cowpea
Source: Plants (Basel). 2024 Aug 15;13(16):2273. doi: 10.3390/plants13162273 (PMC11360203; doi:10.3390/plants13162273)
Supplement: Supplementary file 1 [file plants-13-02273-s001.zip › Figure S1.Phylogenetic tree depicting the evolutionary relationships among WRKY domains of cowpea and soybeanSelected Arabidopsis WRKY genes were used as representatives for analysis..pdf]

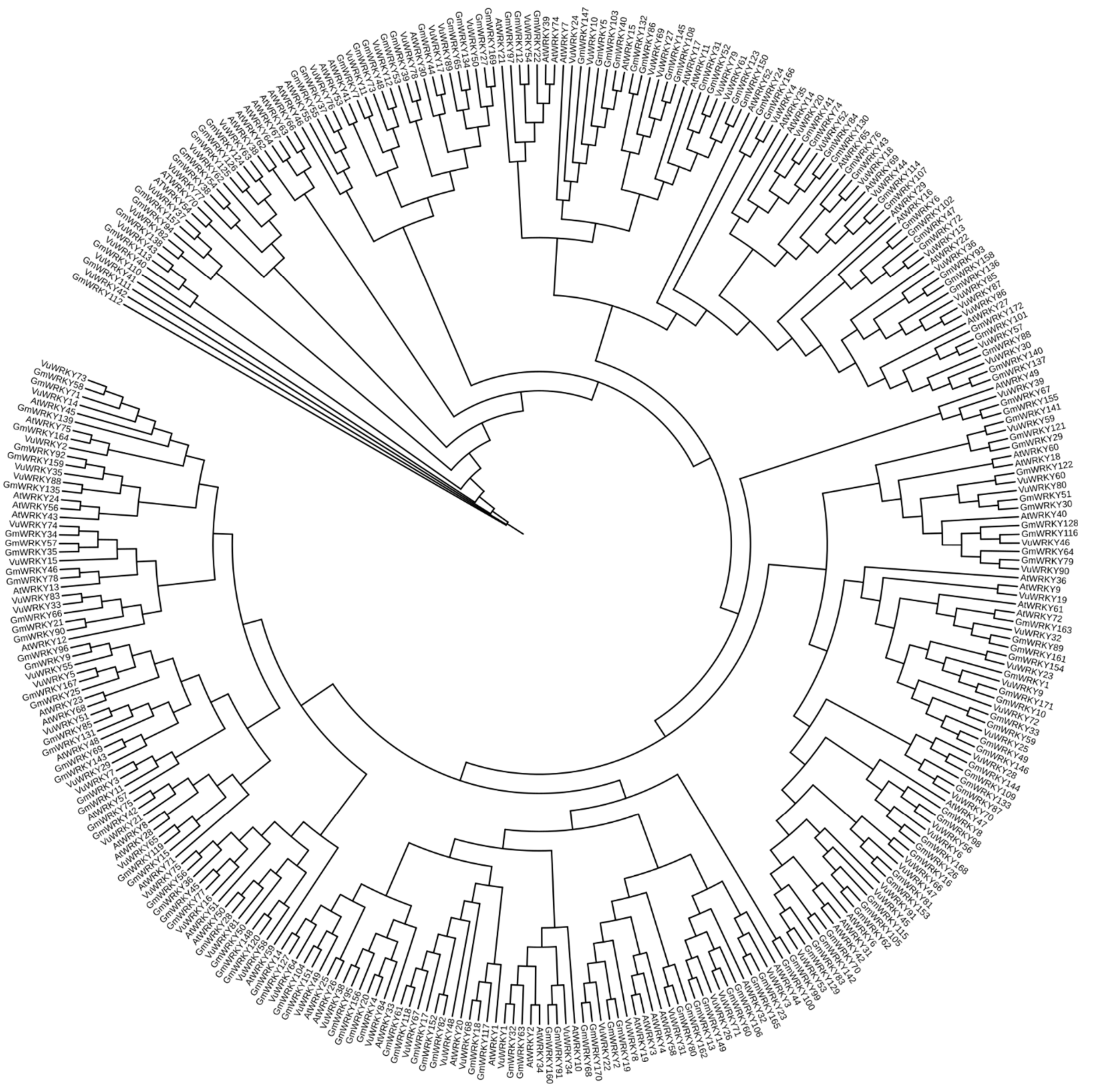

Figure S1. Phylogenetic tree depicting the evolutionary relationships among WRKY domains of cowpea and soybean. Selected Arabidopsis WRKY genes were used as representatives for analysis.
