# Supplementary material for: Identification and Analysis of WRKY Transcription Factors in Response to Cowpea Fusarium Wilt in Cowpea
Source: Plants (Basel). 2024 Aug 15;13(16):2273. doi: 10.3390/plants13162273 (PMC11360203; doi:10.3390/plants13162273)
Supplement: Supplementary file 1 [file plants-13-02273-s001.zip › Figure S2. Alignment of the structural sequences of 91 cowpea (VuWRKY) and 8 Arabidopsis (AtWRKY) WRKY proteins..pdf]

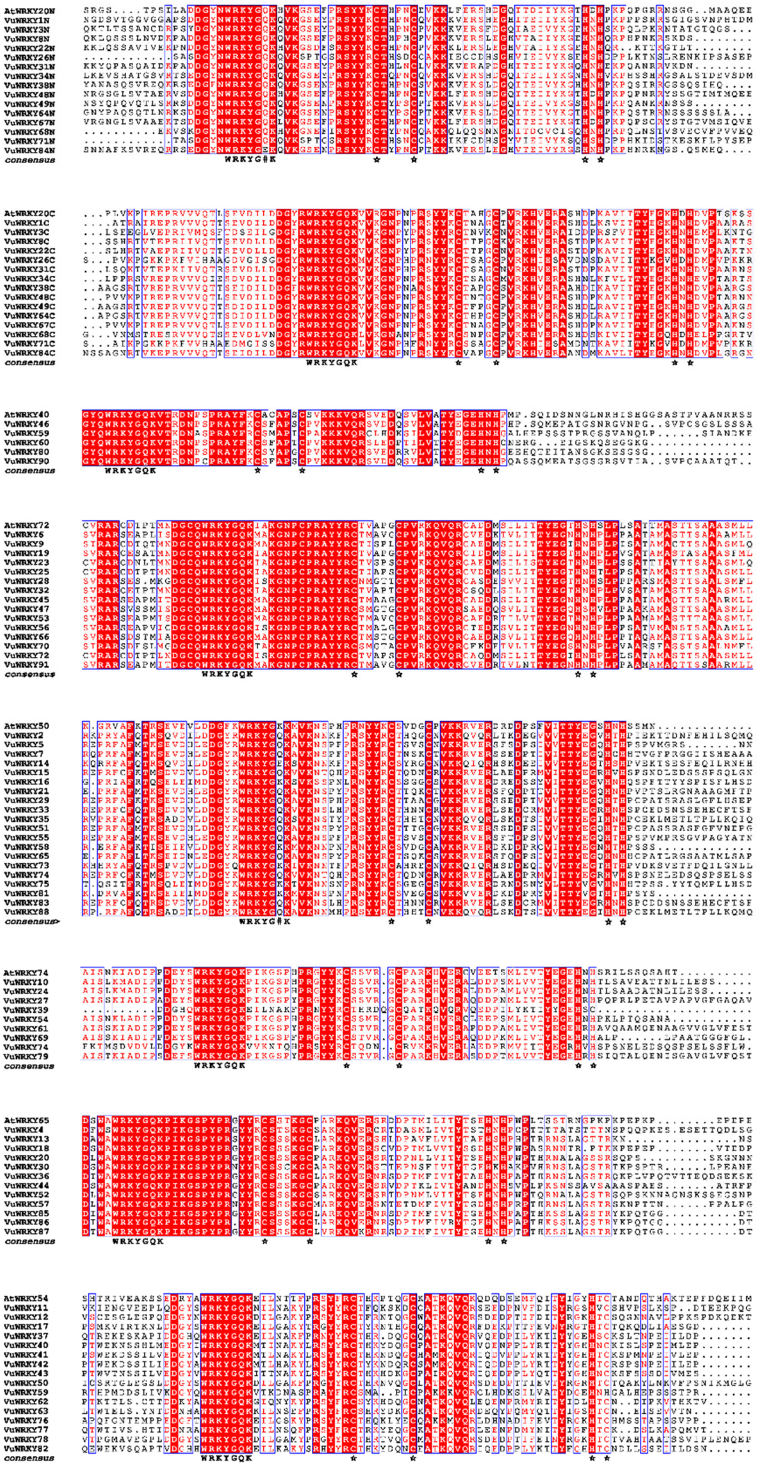

Figure S2. Alignment of the structural sequences of 91 cowpea (VuWRKY) and 8 Arabidopsis (AtWRKY) WRKY proteins.

For WRKY proteins in Group 1, the N-terminal and C-terminal WRKY domains are denoted by "N" and "C", respectively. The WRKY domain is represented by WRKYGQK, with any variations indicated by "#". Typical amino acids in the zinc finger motif are represented by pentagrams.
